# Crayons of Nitrate of Silver and Potassa

**Published:** 1848-03

**Authors:** 


*Crayons of Nitrate of Silver and Potassa.*—We extract the following from the Bulletin General de Therapeutique. According to Dr. Desmarres, sulphate of copper, an efficacious caustic in cases of granulations, while they are vascular, is powerless when they become pale and nearly cartilaginous. On the other hand, cauterization with the pencil of nitrate of silver sometimes causes a too severe reaction, and thus occasions serious accidents. With a view of preventing these two inconveniences, the insufficiency of the sulphate of copper, and the excessive energy of the pure nitrate of silver, M. Desmarres caused a series of crayons to be prepared, composed of nitrate of silver and nitrate of potash, in the proportion of one-half, one-fourth, and one-eighth of the former. They are prepared in the following manner.

The two salts are mixed and fused in a crucible of silver or platinum, and stirred from time to time with a glass rod. As soon as the mass is in tranquil fusion, it is poured into moulds in the same manner as the nitrate of silver. These pencils are hard, firm, smooth, and unalterable in the air, and they can be carried in a case like lunar caustic.—*Dub. Med. Press.*

